# Single-molecule tracking in living microbial cells

**DOI:** 10.52601/bpr.2024.240028

**Published:** 2025-02-28

**Authors:** Xiaomin Chen, Qianhong Guo, Jiexin Guan, Lu Zhang, Ting Jiang, Liping Xie, Jun Fan

**Affiliations:** 1 Institute of Fundamental and Frontier Sciences, University of Electronic Science and Technology of China, Chengdu 611731, China; 2 School of Life Sciences and Engineering, Southwest University of Science and Technology, Mianyang 621010, Sichuan, China; 3 School of Life Sciences and Technology, University of Electronic Science and Technology of China, Chengdu 611731, China; 4 Yangtze Delta Region Institute (Huzhou), University of Electronic Science and Technology of China, Huzhou 313000, Zhejiang, China

**Keywords:** Single-molecule tracking, PALM, Diffusion coefficients, Trajectory, Living microbial cells

## Abstract

Some microbes are referred to as model organisms because they are easy to study in the laboratory and hold the ability to retain their characteristics during DNA replication, DNA transcription, and other fundamental processes. Studying these microbes in living cells via single-molecule imaging allows us to better understand these processes at highly improved spatiotemporal resolution. Single particle tracking photoactivated localization microscopy (sptPALM) is a robust tool for detecting the positions and motions of individual molecules with tens of nanometers of spatial and millisecond temporal resolution *in vivo*, providing insights into intricate intracellular environments that traditional ensemble methods cannot. With this approach, the fluorophores are photoactivated stochastically, a series of images are recorded, and the positions of fluorophores are identified in these images, and ultimately the locations are linked together to yield trajectories of individual molecules. Quantitative kinetic and spatial information, such as reaction rates, diffusion coefficients, and localization maps, can be obtained by further analysis. Here, we present a single-molecule tracking protocol that includes sample preparation, data acquisition and brief data processing. This protocol will enable researchers to directly unveil molecular and cellular mechanisms underlying the essential biological processes.

## INTRODUCTION

Protein–DNA interactions are essential for many fundamental biological processes, such as transcription, replication, repair, and among others. Visualization and quantification of intracellular molecular motions and interactions in living cells provide crucial insights into the molecular and cellular mechanisms underlying biological processes (Banaz *et al.* 2019; Uphoff *et al.* 2014). For decades, varieties of fluorescence imaging methods were put forward to unravel how molecular interactions occur in living cells at high spatiotemporal resolution (Banaz *et al.* 2019; Xue *et al.* 2015). Among these methods, one of the most promising ways for tracking the motions of cytoplasmic proteins is sptPALM, allowing for investigations of the dynamics of individual molecules in living cells.

Due to the diffraction limit, the spatial resolution of conventional optical microscopy techniques is limited to roughly half the wavelength of light, therefore it is difficult to resolve the subcellular organization of individual molecules and molecular complexes that are smaller than this limit (Lelek *et al.* 2021). To break the limit, various super-resolution microscopy methods have been developed, one of which is single-molecule localization microscopy (SMLM) (Zhu *et al.* 2023). Photoactivated localization microscopy (PALM) is an SMLM approach which can sequentially acquire random subsets of fluorophores that alternate between ON and OFF states and determine their positions (Betzig *et al.* 2006). Traditional epifluorescence microscopy illuminates and excites the entire sample with light penetrating more than 1 mm, however, the defocused light emission will generate a substantial background and thus interfere with the fluorescence signal. The highly inclined and laminated optical sheet (HILO) microscopy technique for single-molecule imaging within cells notably enhances the signal-to-noise ratio by illuminating the sample with a highly inclined and thin laser beam, which can penetrate a few microns into the cellular environment, enabling the visualization of fluorophores within the live cells (Tokunaga *et al.* 2008). In addition, for the interest of temporal resolution, it is feasible to go beyond the limit of 100 Hz by defining a smaller region of interest (ROI) and to detect the fluorophore with a modest quantity of emitted photons with the assistance of electron multiplying charge-coupled device (EMCCD) cameras (Bayle *et al.* 2021; Dahal *et al.* 2023).

In order to perform single-molecule tracking inside microbial cells, the proteins of interest (POI) need to be fluorescently labeled at the native chromosomal locus. There are basically two methods to label intracellular proteins: either via internally expressed fluorescent proteins (FPs) such as GFP, PAmCherry, PATagRFP, and mMaple3; or by employing the cell-permeable synthetic dyes like Janelia Fluor (JF) or PA-JF rhodamine dyes coupled to the genetically encoded self-labeling protein tags (SLPs, such as Halo-tag, SNAP-tag and CLIP-tag). The size of the FPs and SLPs sometimes perturb the dynamics of POI. Many factors need to be considered when choosing the optimal labeling strategy for sptPALM. The FPs are prone to fast photobleaching and form dimmer. Since the high copy number of POI makes FPs (such as GFP and RFP) hard to differentiate single molecules due to overcrowding and diffraction limit, photoactivatable (PA)/photoswitchable (PS) FPs were developed to allow for sparse labeling of POIs. mMaple3 seems to be the best choice as it has optimal performance in all four criteria that strongly influence the quality of super-resolution images (Wang *et al.* 2014). Despite its low dimerization tendency, PAmCherry remains a popular FP, with several successful applications of sptPALM in bacteria. Although a few single-molecule tracking investigations have been performed using GFP and its variants in yeast, the micron-scale penetration depth of HILO makes them hardly the optimal option to track the proteins expressed inside the cells with larger sizes (Podh *et al.* 2021), namely the FPs or their PA/PS derivatives FPs may not be appropriate for yeast cells. The synthetic dyes utilized in the second way can be generally brighter with enhanced photostability and flexibility, which yields an immense number of trajectories per cell, regardless of the copy number of POI (Banaz *et al.* 2019; Ha *et al.* 2022). However, compared to FPs, extra operations would be employed to achieve good labeling by the internalized synthetic fluorescent probes, which bind covalently to the SLPs with a 1:1 stoichiometry (Banaz *et al.* 2019). The labeling efficiency can thus be optimized by coordinating the concentration of fluorescent probes and the incubation time (Podh *et al.* 2021; Yan *et al.* 2024). By finely tuning the labeling density, one may realize single-molecule detection even using the standard FPs (like TagRFP-T) or synthetic dyes (like JFX650)(Yan *et al.* 2024). Currently, JF dyes work well in both *E. coli* and yeast (Banaz *et al.* 2019; Nguyen *et al.* 2023). When it comes to binding to JF dyes, the Halo-Tag outperforms SNAP-tag or CLIP-tag for tracking in living cells, displaying higher photostability and less nonspecific binding (Presman *et al.* 2017). Since PA-JF 549 cannot label MukB-Halo efficiently, this series of dyes might not be suitable for *E. coli* or alternative attempts need to be made (Banaz *et al.* 2019). However, PA-JF dyes have been proven to be practicable for tracking in yeast cells (Kapadia *et al.* 2020).

Through surpassing the diffraction limit and reducing the scattered light and background fluorescence, the combination of super-resolution PALM microscopy and single-particle tracking (SPT) method has proven to be highly efficient in facilitating direct visualization of the motions of thousands of proteins inside living cells (Dahal *et al.* 2023; Tokunaga *et al.* 2008), which allow scientists to study the positions, trajectories, and interactions of molecules in complex and dynamic cellular environment *in vivo*. Upon data acquisition, the localizations of individual POIs can be determined with nanometer accuracy using image processing algorithms. Trajectories can be extracted by linking the localizations from the same molecule appearing in adjacent frames with a temporal resolution of milliseconds. The precise localizations, trajectories, and motion behavior of individual molecules allow real-time access to the biophysical parameters regarding biomolecular interactions, such as diffusion coefficient, binding kinetics, sizes of clusters and spatial distributions (Banaz *et al.* 2019). Single-molecule measurements via sptPALM further the bottom-up understanding of the dynamic interactions between DNA and partner proteins and contribute to establishing a precise model of molecular and cellular reactions. Numerous applications of this approach generated instructive perceptions of various fundamental cellular processes in both prokaryotic and eukaryotic cells (Bayle *et al.* 2021; Dahal *et al.* 2023; Ghodke *et al.* 2020; Kapadia *et al.* 2020; Nguyen *et al.* 2021, 2023).

Here, we present a general protocol for sptPALM in living microbial cells, including three steps: sample preparation, data acquisition, and data processing. We demonstrate how to perform single-molecule tracking in living *E. coli* and yeast cells allowing for the quantification of molecular dynamics, interactions and kinetics. Through this protocol, we hope to provide a framework for more *in vivo* single-molecule imaging studies in other microbial cells and even eukaryotic cells.

## PROTOCOL OVERVIEW

**Figure 1:**
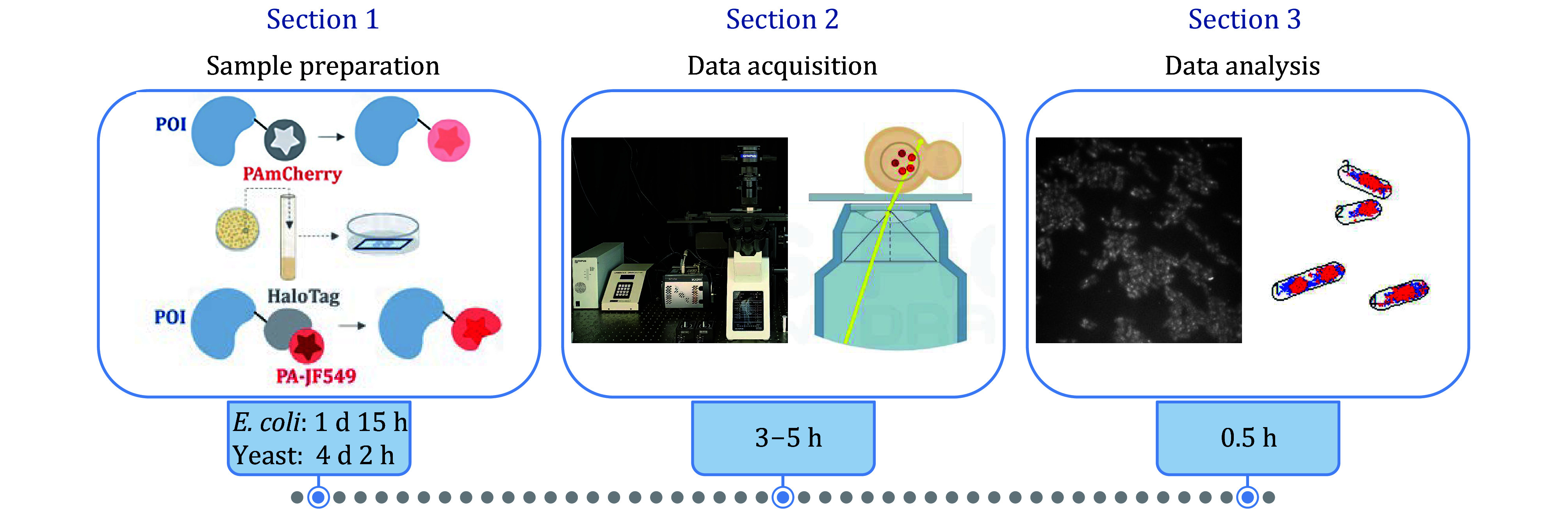
General schematic of the protocol

As shown in Fig. 1, this protocol overall consists of three sections. In brief, the first section provides a detailed procedure of sample preparation for single-molecule imaging. POIs are subjected to fluorescent labeling firstly using classic molecular biology methods. Usually, a fluorescent protein or an SLP tag is genetically inserted into the endogenous locus at the N- or C-terminus of the target gene. Next, test if the labeling has an impact on the function of POI by checking the doubling time of cell growth or by checking the cell shapes under a microscope. The labeled cells are then cultured in the appropriate medium and harvested when reaching at the exponentially-growing state, after which the cells are incubated with proper dyes for SLPs labeling according to the statements from manufacturers. Then the labeled samples are collected for microscopy imaging by using agarose pads or coated coverslips. In the second section, a detailed guideline for microscopy data acquisition is provided with the tricky steps, such as tuning the power of 561 nm laser light to allow limited number of fluorescent molecules excited in individual cells and adjusting the incident angle to get the HILO illumination of the fluorescent signals in the field of view (FOV) with a good cell density. In the third section, we provide a brief description of data processing for sptPALM. A Gaussian fitting can be applied to identify the localizations of fluorescent molecules from band-pass filtered data. To acquire the trajectories of individual molecules, the molecular localizations appearing in successive frames are linked based on their proximity. The prevalent diffusive states, abundance, subcellular distributions of these molecules are then determined by analyzing the molecular trajectories.

## MATERIALS

Reagents and materials, and the equipment used in this work are listed in Table 1 and Table 2.

**Table 1:** Reagents and materials

Reagents and materials	Source	Identifier
Trypton	OXOID	#LP0042B
Yeast extract	OXOID	#LP0021B
NaCl	Sinopharm Chemical Reagent	#7647-14-5
Agar powder	Solarbio	#ST004D
Poly-L-lysine	Sangon Biotech	#A600751-0025
Bacto-agar	OXOID	#LP0011B
D-glucose	KESHI	#14431-43-7
Agarose	Bio-RAD	#1613103
PA-JF 549	Tocris Bioscience	#1811539-42-0
Concanavalin A from Canavalia ensiformis	Sigma Aldrich	#11028-71-0
SD medium	Coolaber	#PM2101
PBS	Biosharp	#BL302A
Cover glasses	VWR	#631-0172
Gasket	Grace Bio-Labs	#103280

**Table 2:** Equipment

Equipment	Source	Identifier
405 nm laser	CNI Laser	MDL-III-405
561 nm laser	Coherent	OBIS LS 561 nm 80 mW
Inverted microscope	Olympus	IX83
Dichroic mirrors	Chroma	ZT405/488/561rpc
Oil-immersion objective lens	Olympus	UPLAPO100XO
Multiple band filter	Chroma	ZET405/488/561x
EMCCD	Andor	IXON-L-897
Long-pass filter	Chroma	ET575lp
LED light	Philips	253.7nm×20W
Collimated LED light	Thorlabs	M660L4-C1
100× objective	Olympus	UPLAPO100XO
Motorized piezoelectric stage	ASI Imaging	MS-2000
Z-motor objective mount	ASI Imaging	PZ-2000FT

**[Note]** Since the single-molecule tracking experiments are basically carried out on a TIRF-based microscope, hereby, the above-mentioned lasers, microscopes, and cameras from other manufacturers with comparable power intensities, stabilities and performances can be employed according to the users' choices and affordability.

## PROCEDURE

### Sample preparation for single-molecule imaging

#### Sample preparation of E. coli cells

##### Cell culture

**Figure 2:**
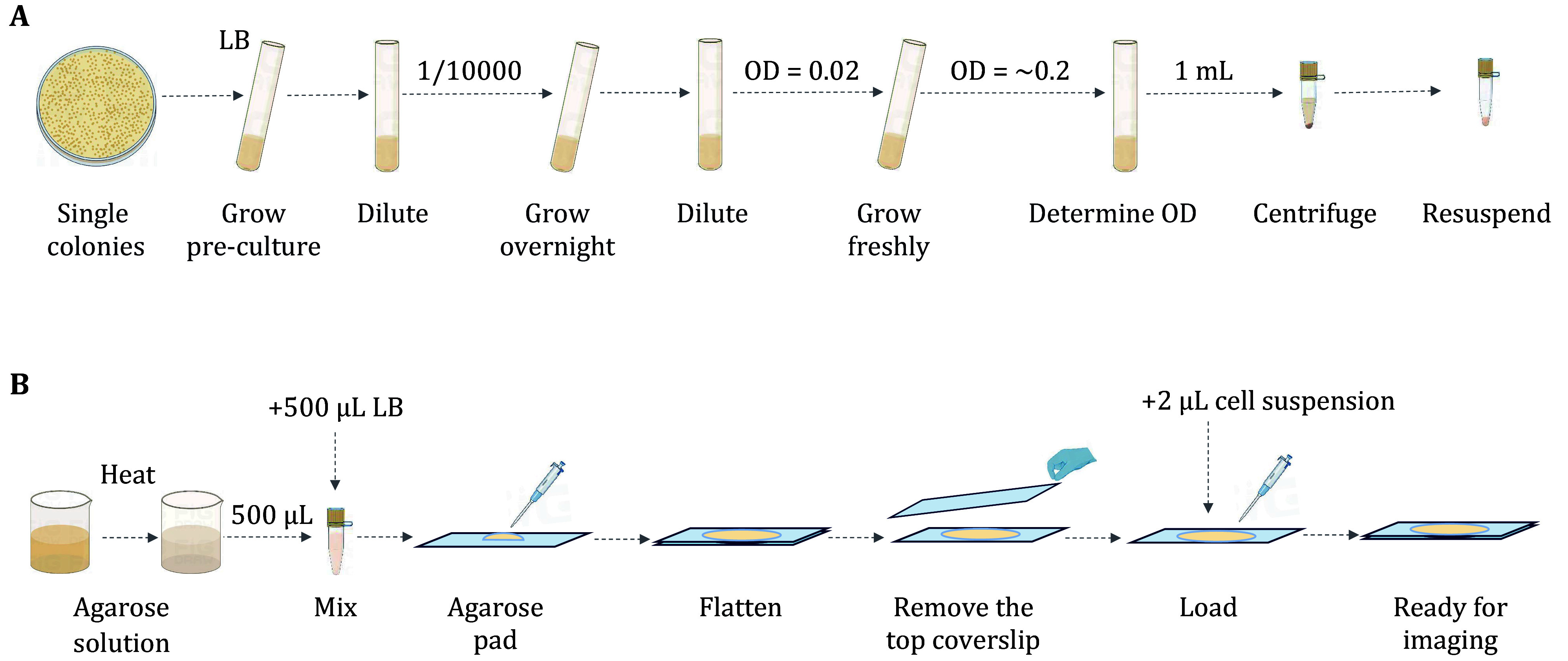
Illustration of steps for culturing *E. coli* for single-molecule imaging. **A** After cultivating *E. coli* to the early exponential growth stage, centrifuge and resuspend the cells. **B** Preparation of the microscope slides by agarose pad

The *E. coli* strain carries a fusion of N- or C-terminal PAmCherry/mMaple3 with the target protein. Fluorescence tagging experiments were performed in *E. coli* by placing the FP either after the promoter or before the terminator of the target gene by lambda-Red recombination (Sharan *et al.* 2009). Here, the fusion of PAmCherry with the C-terminus of RNAP (RNA polymerase) in MG1655 is taken as an example in this protocol. Cellular growth rate is then determined to confirm the function of co-expressed proteins. More details about strain construction can be found in other well-written references (Datsenko and Wanner 2000; Reyes-Lamothe *et al.* 2008; Uphoff *et al.* 2013). The detailed steps are shown in Fig. 2.

(1) On Day 1, streak the PAmCherry-labeled *E. coli* strain from the glycerol stock onto a Luria Broth (LB) plate supplemented with 25 μg/mL kanamycin and incubate overnight at 37 °C.

(2) In the morning of Day 2, inoculate a 5 mL LB culture from a single colony and incubate at 37 °C for 6–8 h with shaking at 220 r/min.

(3) Dilute the above pre-culture product 1:10,000 into 5 mL of LB and incubate overnight at 37 °C with shaking at 220 r/min.

**[Note]** The dilution step is mainly for switching the culture media after the inoculation, for instance, diluting the LB culture product into an M9 medium growing state. If the culture medium stays as the same, this step can be ignored sometimes. Without changing the LB media, we still place this step here just to indicate one step for some potential reinoculation into different culture media.

(4) In the morning of Day 3, determine the optical density 600 (OD, for short hereafter) spectrophotometrically and dilute the culture into 5 mL of fresh LB medium to OD = 0.02. Grow at 37 °C with shaking at 220 r/min for 1.5 h to an OD of 0.1–0.2.

**[Note]** Researchers should culture the cells at an appropriate OD/growth state based on the biological question, regardless of the species of microbe. M9 or EZRDM is more suitable for single-molecule imaging with lower fluorescence background. Imaging with the LB-based agarose pad is still achievable based on our experiences, however, it may cost a bit longer to do the photobleaching procedure prior to the activation steps by 405-nm laser.

(5) Concentrate 1 mL of cell culture by centrifugation at ~600 *g* for 5 min in a 1.5 mL microcentrifuge tube. Remove the supernatant and resuspend the cell pellet with 20 µL fresh LB medium by mechanical pipetting.

**[Note]** To disperse the cells, the pellet should be resuspended by pipetting thoroughly.

##### Microscope slide preparation

(1) Prepare 5 mL of 2% (*w*/*v*) low-fluorescence agarose solution with ddH_2_O in a 10 mL beaker. Melt the agarose with a microwave oven till the mixture turns clear. Mix 500 μL of the melted agarose solution with 500 μL of 2× LB by pipetting.

**[Note]** Remember to use gas permeable sealing film to prevent significant changes in agarose concentration. The 1 mL pipette tips for mixing should be cut short the sharp end by 0.5 cm.

(2) Put ~500 μL of the hot mixture in the middle of the coverslip and flatten the pad with another coverslip. This should be done within 1–3 min.

**[Note]** This step must be carried out rapidly before the mixture cools down. The coverslips used here need to be burned at 500 °C with a furnace for an hour to remove the background fluorescence sources in advance. Covering in clean aluminum foil, burned coverslips can be kept at room temperature for several weeks. Instead of burning, some other cleaning methods can also be employed to clean the coverslips, such as chemical methods or plasma cleaning (Thanu *et al.* 2019; Yan *et al.* 2024).

(3) Remove the top slide from the agarose pad after the mixture cools down, add 2 μL of the resuspended cell onto the pad. Cover back the aforementioned slide onto the pad after several minutes. The cells are ready for imaging. This step may take 3–5 min.

**[Note]** Try to evaporate the water in the cell culture solution as much as possible, otherwise the cells will move towards the edge of the pad. The cells should be imaged within 45 min before the agarose pad dries.

#### Sample preparation for Saccharomyces cerevisiae cells

##### Cell culture

**Figure 3:**
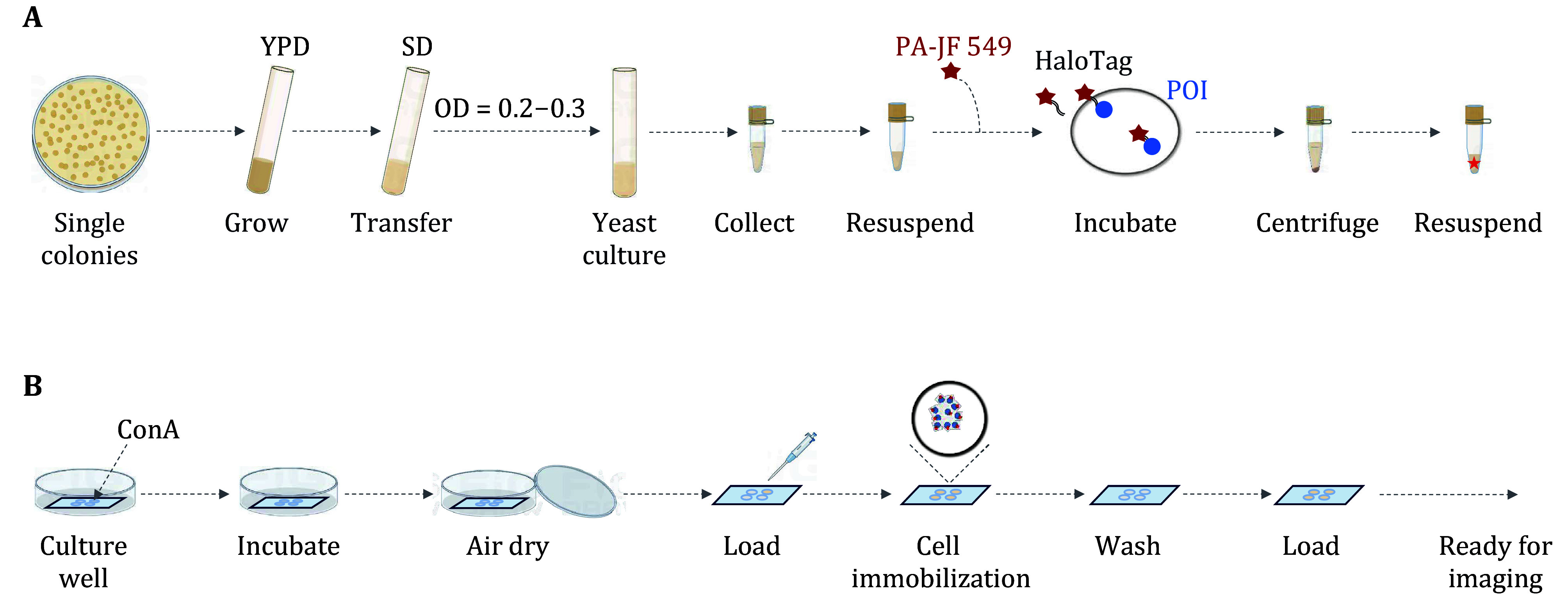
Procedure for labeling Halo tag with a fluorescent ligand in living yeast cells. **A** Culture the yeast cells to the exponential growth stage. **B** Coat the coverslips with ConA and prepare the imaging sample

To perform single-molecule imaging inside living yeast cells, the *PDR5* gene should be deleted firstly by replacing it with a KanMX cassette, after which the SLPs-Halo tags are supposed to be inserted at the N/C terminus of the endogenous locus of the POIs. PDR5 is a plasma membrane-bound ABC transporter involved in actively exporting the dyes from the cell. The yeast strains bearing *pdr5*Δ enhance the retention of dyes, allowing the principal labeling of the majority of POIs (Brouwer *et al.* 2020). Scarless gene tagging in *Saccharomyces cerevisiae* can be achieved through one-step transformation and two-step selection (Landgraf *et al.* 2016). Here, the yeast strain of BY4741 (RPB1-Halo, *pdr5*Δ) is taken as an example. The detailed steps are shown in Fig. 3.

(1) On Day 1, streak the BY4741 cells from the stock onto a YPD (Yeast Extract Peptone Dextrose Medium) plate and incubate at 30 °C for three days to obtain the colonies.

(2) In the morning of Day 4, pick up a colony and inoculate it into 5 mL of SD medium. Grow at 30 °C for 12 h with shaking at 250 r/min.

(3) In the evening, inoculate 1 μL cell cultures into 5 mL fresh SD medium and grow at 30 °C for 12 h till OD reaches 0.2–0.3.

**[Note]** If it’s uncertain how fast the yeast strain grows, make a dilution series.

(4) On Day 5, measure the OD with a spectrophotometer. Spin down 1 mL of cell culture at 1100 *g* for a minute in a 1.5 mL microcentrifuge tube.

**[Note]** If the cells are not so transparent post centrifugation, remove half of the supernatant and repeat the previous step till the cell pellet becomes clear.

(5) Remove the supernatant and resuspend the cell pellet with 500 μL of prewarmed SD medium. Then add PA-JF 549 dye into the culture to a final concentration of 50 nmol/L. After wrapping the centrifuge tube with tinfoil, shake the cells at 30 °C and 250 r/min for 40 min.

**[Note]** The incubation time and the dye concentration necessary for labeling depend on the abundance of the POIs. It’s highly suggestive to titrate different combinations of conditions to achieve the ideal labeling.

##### Sample preparation using concanavalin A

The yeast cell wall is substantially abundant in polymannose carbohydrates that concanavalin A (ConA) avidly binds to. ConA was proven to be superior to poly-L-lysine for adhering the yeast cells to glass slides, with little effect on cell proliferation and division kinetics (Caloca *et al.* 2022). Here, we prefer ConA to immobilize yeast cells.

(1) Put a gasket on the cleaned coverslip. Filter 1 mg/mL of ConA by using a 0.22 μm filter. Pipet 30 μL of the ConA into each well of the gasket. Put the above coverslip into a Petri dish, then cover the dish with the lid. Incubate at about 20 °C for 20 min. Drain the wells and air dry for 20 min at the same temperature with the Petri dish open. Coated coverslips can be stored at 20 °C for several months. All of the procedures are carried out at room temperature unless otherwise specified.

(2) After incubation with the dye, collect the yeast cells and wash with 1 mL fresh SD medium for five times to remove the free dye. Spin down the cells and resuspend the cell pellet into 100 μL SD medium.

(3) Pipet 30 μL of yeast cells into the ConA-coated well and incubate for 20 min at 30 °C.

(4) Rinse the cells by adding 30 μL of prewarmed SD and get rid of the medium. Repeat the rinse step twice.

(5) Add 30 μL of prewarmed SD into the well for imaging.

**[Note]** Yeast cells can be imaged within 15 min before evaporation, otherwise the cells will shrink.

#### Data acquisition

Total internal reflection fluorescence microscopy (TIRF) has been usually employed to enhance the signal-noise-ratio of single-molecule imaging by restricting the excitation and detection of fluorophores into a thin layer above the coverslip surface. Due to the thickness of microbial cells, single-molecule imaging in this protocol requires highly inclined illumination, namely HILO microscopy, which can be achieved on a TIRF microscope by slightly reducing the angle of the excitation light. Imaging intracellular proteins in *E. coli* requires an illumination depth of ~0.8 µm, while yeast cells require a deeper illumination region of up to several microns. Here, a 405-nm photoactivation laser and a 561-nm excitation laser are required for PALM imaging with PAmCherry and PA-JF 549, as we present the overall procedure for obtaining a PALM video allowing to track single molecules.

(1) Clean a microscope objective and add a drop of immersion oil onto the objective.

(2) Place the coverslip with cells onto the microscope stage to end up with the cells-mounted coverslip touching the objective.

(3) In the Andor Solis software, typical settings can be loaded through the saved configuration file. Define an appropriate FOV to guarantee the ideal frame rate for tracking individual molecules with certain molecular weights and to achieve the proper read-out speed of the camera. An exposure time of 17.46 ms/frame (including 1.76 ms camera readout) was set for the tracking of both RNAP in *E. coli* and RPB1 in yeast. Imaging data should be saved in fits/tiff format for further localization analysis using ImageJ and MATLAB.

**[Note]** When choosing the excitation power and exposure duration, a balance between low photobleaching/phototoxicity and high signal-to-noise must be established. Furthermore, the mobility of POI influences the selection of the optimal exposure duration and excitation power. The faster the protein moves, the shorter exposure time and stronger excitation power are required. For diffusing protein, long exposure times for protein diffusion will cause motion blur, hence exposure times of 10–30 ms and excitation power of 5–10 mW are advised.

(4) Set the shutter to a bright field and switch on the LED illumination, and the intensity of LED needs to be adjusted for imaging. Find a suitable FOV and identify the cells in the focal plane by tuning the coarse and fine adjustment knob. Record a short video of the bright field images with a good density of microbial cells.

**[Note]** To protect the camera from overexposure, the EMCCD camera gain must be switched off when the brightfield LED illumination is on.

(5) Switch off the LED light and switch on the EMCCD camera gain. Set the shutter to 561-nm channel. Switch on the 561-nm laser until there is almost no pre-activated fluorescence or background fluorescence signal (approximately 1–3 min). During photobleaching, the angle of the excitation beam requires to be adjusted to illuminate the cells evenly. To this end, the 561-nm laser beam is focused into the back focal plane of the 100× NA 1.5 objective in HILO mode. The inclined illumination should be adjusted well with an incident angle of around 60°–65°, which allows for an illumination beam passing through a good depth of the cell samples. One may refer to the original work by Tokunaga et al for a better understanding of incident angle for HILO illumination (Tokunaga *et al.*^2008^).

**[Note]** Intense illumination can be phototoxic to the cells so the pre-bleaching procedure should be controlled at the minimum level. An excitation power of 5–10 mW is required for several minutes until there is no obvious fluorescence signals in the cells.

**Figure 4:**
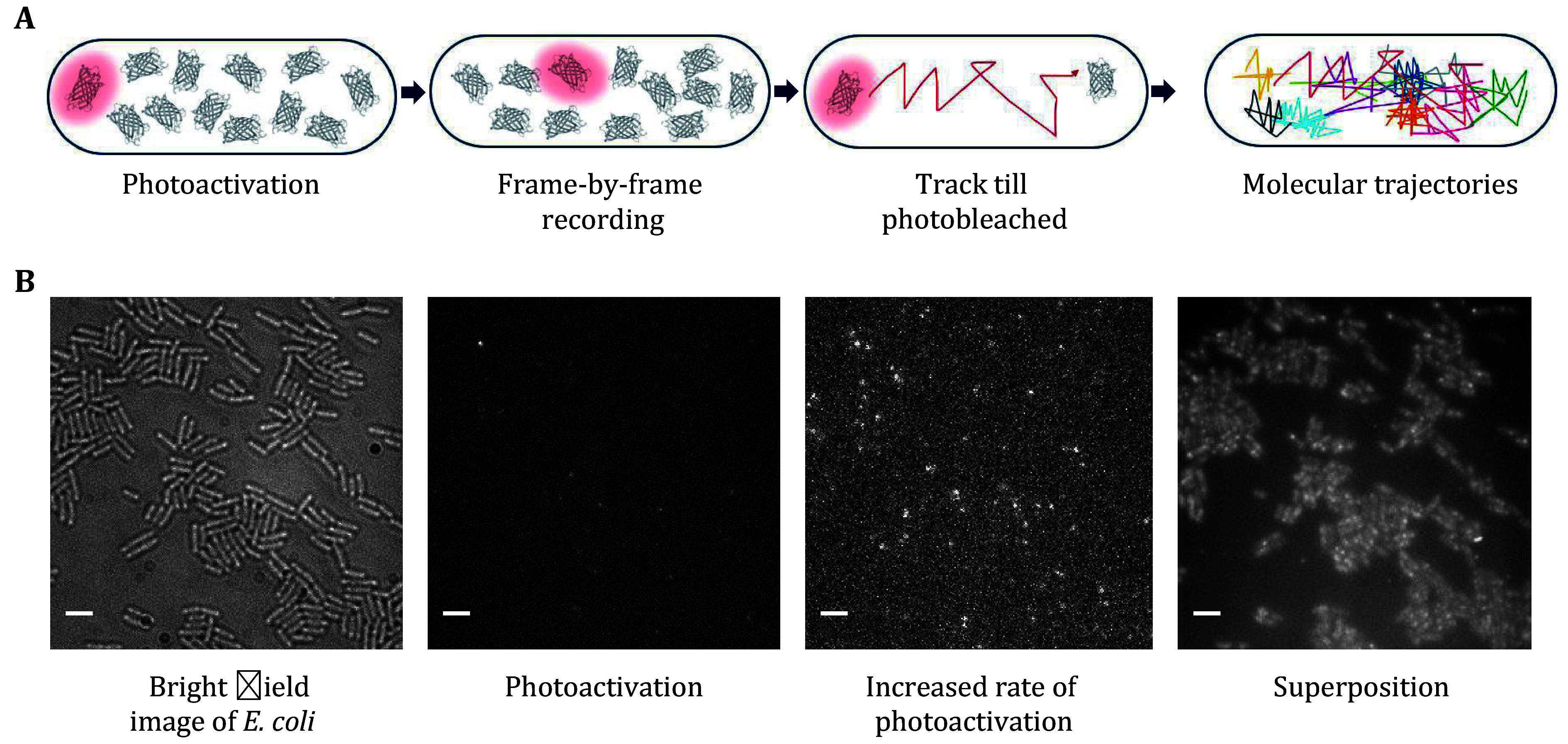
Photoactivation and tracking of RNAP–PAmCherry fusion proteins in living *E. coli* cells. **A** The concept of sptPALM. PAmCherry can be photoactivated from an initial nonfluorescent state upon irradiation with 405 nm light. Track the fluorophore at a rate of several tens of frames per second for several frames until photobleaching. Tracks of many molecules are recorded in a sequential manner. **B** Representative results from a PALM movie of RNAP-PAmCherry. Transmitted light microscopy images of cells immobilized on an agarose pad are taken first. Photoactivate a single PAmCherry in a cell. A higher photoactivation rate increases the number of PAmCherry. Integrated PAmCherry fluorescence from a PALM movie. Scale bar: 3 μm

(6) Switch on the 405-nm laser to stochastically activate a limited number of PAmCherry/PA-JF549 fluorophores for each cell per frame by optimizing a low level of 405-nm laser at a power of 0.02–0.08 W/cm^2^. Empirical excitation power is 0.075 kW/cm^2^, which permits sufficient signal and minimal photobleaching of fluorophores (Fig. 4A). During imaging, the focus of fluorophores and the incident angle need to be well-tuned depending on the fluorescence signal. Record the PALM movie under continuous 561-nm excitation with EMCCD gain.

(7) Record tens of thousands of frames per movie till almost all of the fluorescence signals are photoactivated and captured (about 10 min). Repeat the acquisition procedure for multiple FOVs (Fig. 4B).

**[Note]** Each FOV can be imaged only once since PAmCherry/PA-JF 549 fluorophores are photoactivated and bleached irreversibly.

#### Data analysis

The PALM movies are subsequently analyzed by a custom software written in MATLAB (Fan *et al.* 2023; Stracy *et al.* 2015; Uphoff *et al.* 2013). Here is just a brief introduction to the data analysis. For more details, please refer to other publications (Uphoff *et al.* 2013, 2014).

(1) The candidate fluorophores are identified in band-pass filtered images with a 7 × 7 Gaussian Kernel by a local maximum search. The point spread function (PSF) often shows aberration away from the focal plane and the elliptical Gaussian PSF model is sufficient to achieve best-in-class performance at low spot density for the 2D images. Some other non-Gaussian PSF models may be adopted if the more complex imaging analysis, such as biplane or astigmatic models (Sage *et al.* 2019). Here, the PSF of a single fluorophore can be described by elliptical Gaussian function (see equation 1), which contains a total of seven free fit parameters (x-position (*x*), y-position (*y*), x-width (*σ*_*x*_), y-width (*σ*_*y*_), rotation angle (*θ*), amplitude (*A*), and background offset (*B*)). Then the locally-brightest pixel position is determined to serve as the initial guess for fitting with the elliptical Gaussian function to obtain the precise localization of fluorophores. Details of the algorithms can be found in other references (Holden *et al.* 2010; Wieser and Schütz 2008).

1 $ I\left( {x,y} \right)  =  A{e^{ - \left( {\tfrac{{{{\left( {\left( {x \,-\, {x_0}} \right)\cos \theta \,-\, \left( {y \,-\, {y_0}} \right)\sin \theta } \right)}^2}}}{{2\sigma _x^2}} + \tfrac{{{{\left( {\left( {x - {x_0}} \right)\sin \theta \,+\, \left( {y \,-\, {y_0}} \right)\cos \theta } \right)}^2}}}{{2\sigma _y^2}}} \right)}} + B $

Where *x*_0_ and *y*_0_ are the centroid location of the fluorophore.

**[Note]** Plot the resulting localizations from the whole PALM movie superimposed on the transmitted light microscopy image of the same FOV. Determine the appropriate threshold to achieve the correct detection of fluorophores inside the cells and the majority of background signal can be eliminated (Zhai *et al.* 2023).

**Figure 5:**
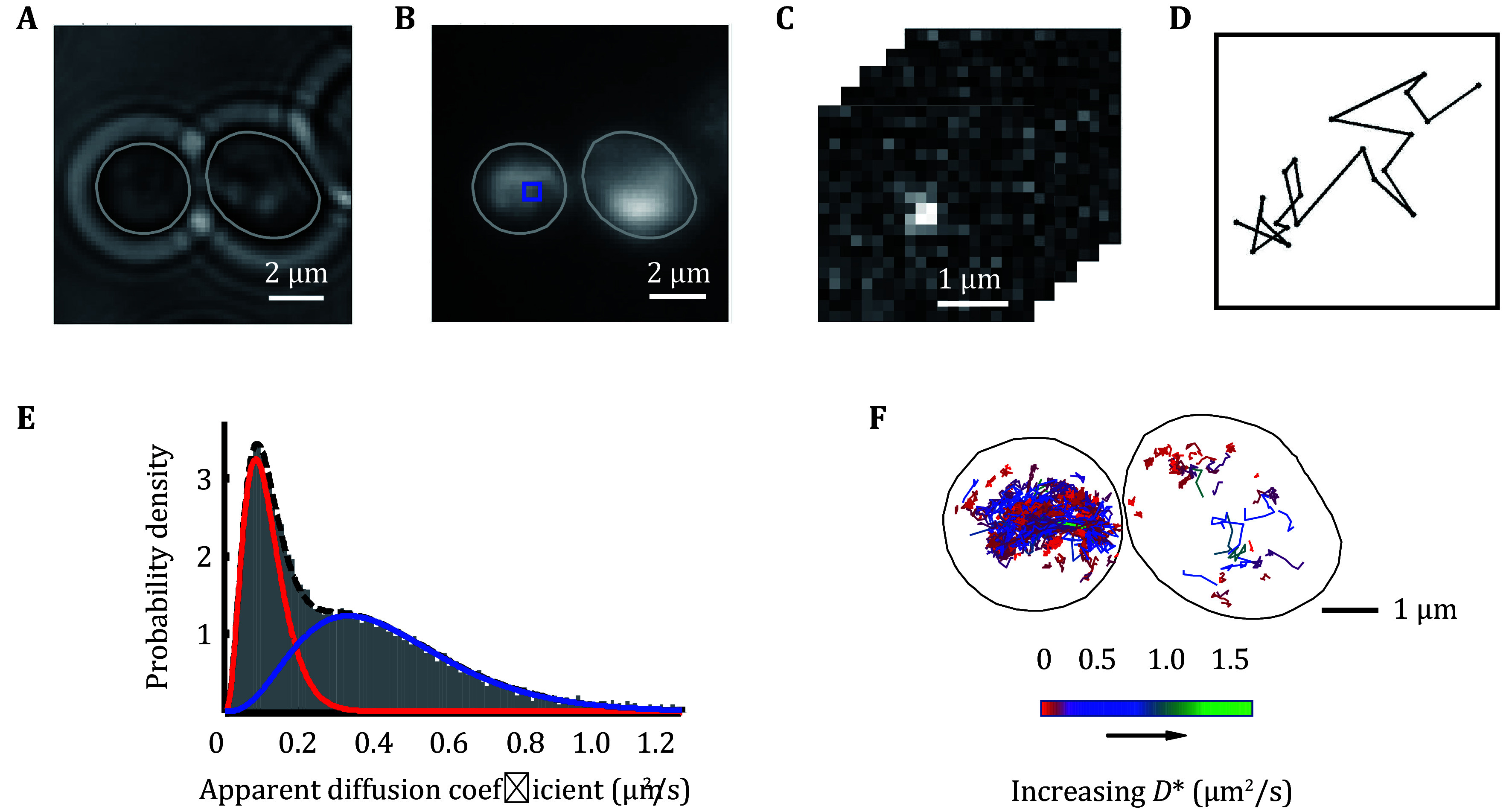
Single-molecule imaging of RPB1-Halo in living yeast cells. **A** Transmitted light microscopy image. **B** Integrated PA-JF 549 fluorescence from a PALM movie of the same FOV. **C** Selected 150 ms frames of raw data from the molecular trajectory shown in the box of Panel B. **D** Trajectory of the single photoactivated PA-JF 549 shown in Panel C. **E** Apparent diffusion coefficient (D*) distribution of the several thousands of tracked RPB1-Halo. F Single-molecule trajectory map of RPB1-Halo in living yeast cells

(2) For automated tracking analysis, nearest-neighbor algorithms are commonly employed. Localizations appearing in consecutive frames within a custom-defined tracking window (3 × 160 nm) are connected to form a trajectory (Figs. 5A–5C). One-frame memory parameter is allowed to minimize the transient loss of fluorophore within a trajectory due to blinking or loss of focus. More details can be found in the references (Uphoff *et al.* 2014; Wieser and Schütz 2008).

(3) To analyze the diffusion behavior of the PAmCherry/Halo-tag fusion protein, the mean-squared displacement (MSD) between successive localizations for each trajectory can be computed using Eq. 2. Short trajectories (*e*.*g*., <4 steps) generally introduce statistical uncertainties in MSD analysis and are usually discarded. To classify the molecular motion by the shape of the MSD curve, plot the curve of MSD over a range of lag time (Δ*t*) that is calculated from multiple frames (Fig. 5D).

2 $ MSD = 1/\left( {N - 1} \right)\sum\nolimits_{i \,=\, 1}^{N \,- \,1} {\left( {{{\left( {{x_{i \,+\, 1}} - {x_i}} \right)}^2} + {{\left( {{y_{i\, + \,1}} - {y_i}} \right)}^2}} \right)} $

(4) The apparent diffusion coefficient D* can be calculated from the MSD, the localization error (here, *σ*_loc_ = 30 nm) is expressed as a positive offset in the D* (*σ*_loc_^2^/Δ*t*). To obtain the diffusion coefficient of different motion species with different rates, fit D* to an analytical equation that contains the information of distribution (see Eq. 3) (Zhai *et al.* 2023). The more trajectories, the better for fitting (Figs. 5E and 5F).

3 $ {D^ * } = MSD/\left( {4\Delta t} \right) - \sigma _{loc}^2/\Delta t $

## CONCLUSION AND DISCUSSION

Here, we provide a step-by-step protocol for single-molecule imaging inside microbial cells and briefly describe the data analysis of sptPALM. Minor adjustments to this protocol are still required depending on the POIs and relevant scientific questions, besides the investigations on some other microorganisms with autoblinking have not been mentioned here (Zhai *et al.* 2023). Rich medium (such as LB and YPD) typically contains autofluorescence components, producing spontaneous fluorescence and leading to more background signal, while minimal medium (such as M9 medium) appears very little fluorescence background (Brouwer *et al.* 2020; Uphoff *et al.* 2014). The cellular autofluorescence and background spots need to be photobleached carefully prior to the acquisition of freshly activated fluorescence signals.

As for cell sample preparation for single-molecule imaging, both *E. coli* and yeast cell samples can be prepared either by using an agarose pad, which is convenient and straightforward to make, or by poly-L-lysine/ConA/Chitosan coated coverslips, which is feasible to combine with microfluidics, allowing for the treatment of chemicals. Poly-L-lysine-coated coverslips are commonly employed for immobilizing *E. coli*, whereas ConA-coated coverslips tend to be better for fixing yeast cells (Benn *et al.* 2019; Rines *et al.* 2011; Wang *et al.* 2019). Although poly-L-Lysine generates cell envelope stress and has been shown to reduce membrane potential in several bacteria (Tréguier *et al.* 2019), Wang *et al*. found that the poly-L-lysine-coated protocol did not affect cell proliferation and division kinetics, but for real-time environmental perturbation experiments, the chitosan-coated protocol is more suitable (Calkins *et al.* 2023; Wang *et al.* 2019).

For single-molecule imaging, there are two ways to fluorescently label the intracellular proteins in microbial cells: (1) by fusion of the POIs with FPs and (2) using synthetic fluorophores to covalently bind to fused SLPs (Banaz *et al.* 2019; Podh *et al.* 2021). The second approach typically produces single-molecule signals of higher quality, although membrane permeability must be taken into account (Podh *et al.* 2021). The choice of FP and dyes depends on the microscope settings, especially the lasers and filters available. For microbial cells with enormous diameters, FPs may not be appropriate for single-molecule imaging of intracellular proteins, particularly for abundant proteins. Just like the yeast cells, there are very few examples of employing FPs for single-molecule imaging of intracellular proteins (Podh *et al.* 2021). Despite the advance in visualizing dynamics of gene-specific chromatin and interacting factors, sparse labeling continues to hinder the *in vivo* studies of protein–protein interaction (Lionnet and Wu 2021). Because the interpretation of sptPALM data can be influenced by multiple reasons (such as the biophysical hypotheses, theoretical simulations, and large size of SLPs) that complicate the biological conclusions, more rigorous algorithms will be required to avoid some possible artifacts (Lionnet and Wu 2021).

## Conflict of interest

Xiaomin Chen, Qianhong Guo, Jiexin Guan, Lu Zhang, Ting Jiang, Liping Xie and Jun Fan declare that they have no conflict of interest.
